# High MYC Levels Favour Multifocal Carcinogenesis

**DOI:** 10.3389/fgene.2018.00612

**Published:** 2018-12-11

**Authors:** Manuela Sollazzo, China Genchi, Simona Paglia, Simone Di Giacomo, Annalisa Pession, Dario de Biase, Daniela Grifoni

**Affiliations:** Cancer Evolution Laboratory, Department of Pharmacy and Biotechnology, University of Bologna, Bologna, Italy

**Keywords:** MYC, field cancerisation, multifocality, *Drosophila*, TSGs, cell competition

## Abstract

The term “field cancerisation” describes the formation of tissue sub-areas highly susceptible to multifocal tumourigenesis. In the earlier stages of cancer, cells may indeed display a series of molecular alterations that allow them to proliferate faster, eventually occupying discrete tissue regions with irrelevant morphological anomalies. This behaviour recalls cell competition, a process based on a reciprocal fitness comparison: when cells with a growth advantage arise in a tissue, they are able to commit wild-type neighbours to death and to proliferate at their expense. It is known that cells expressing high MYC levels behave as super-competitors, able to kill and replace less performant adjacent cells; given MYC upregulation in most human cancers, MYC-mediated cell competition is likely to pioneer field cancerisation. Here we show that MYC overexpression in a sub-territory of the larval wing epithelium of *Drosophila* is sufficient to trigger a number of cellular responses specific to mammalian pre-malignant tissues. Moreover, following induction of different second mutations, high MYC-expressing epithelia were found to be susceptible to multifocal growth, a hallmark of mammalian pre-cancerous fields. In summary, our study identified an early molecular alteration implicated in field cancerisation and established a genetically amenable model which may help study the molecular basis of early carcinogenesis.

## Introduction

The molecular events underlying cancer initiation are largely unknown. It is commonly accepted that most cancers are monoclonal in origin, evolving from a single cell whose lineage accumulates in time multiple molecular insults (Michor et al., 2004; Vogelstein et al., 2013; Feinberg et al., 2016). In particular, driver mutations, which provide cells with a growth advantage and are positively selected during lineage evolution, are generally associated with clonal expansion and are frequently found in pre-malignant lesions (Maley et al., 2004; Lawrence et al., 2014; Curtius et al., 2017). In the 1950s, Slaughter introduced the concept of “field cancerisation”: while studying oral cancers, he observed that they recurred more frequently adjacent to a resected tumour (Slaughter et al., 1953). Therefore, field cancerisation was defined as the process leading to the formation of a tissue sub-territory which, despite a normal appearance, bears a series of alterations that make cells more susceptible to malignant transformation than wild-type neighbours, giving rise to multifocal cancers (Wodarz et al., 2004). Successive studies, also fostered by the development of post-genomic technologies (Metzker, 2010), have demonstrated that this phenomenon is not specific to the oral mucosa, being rather a common feature of epithelial organs (Braakhuis et al., 2003; Dakubo et al., 2007; Nonn et al., 2009; Zeki et al., 2011; Jakubek et al., 2016; Lu et al., 2016; Park et al., 2016; Abdalla et al., 2017; Castven et al., 2017).

Although the interest in deciphering cancer’s molecular signature is obvious, it may be quite difficult to understand what mutations favour and maintain the malignant phenotype: a surprising number of driver mutations is indeed present in pre-cancerous tissues, also in those that are not likely to evolve into a frank malignancy (Hofstad et al., 1996; Martincorena et al., 2015; Kato et al., 2016). This suggests that several alterations are evolutionarily neutral and do not impact cell’s phenotype, maybe depending on their temporal occurrence (de Bruin et al., 2014), the tissue context (Galandiuk et al., 2012; Gagneur et al., 2013; Vermeulen et al., 2013) and the genetic background (Chandler et al., 2013). In human tissues, a number of genetic alterations have been associated with field cancerisation (Papadimitrakopoulou et al., 1996; Braakhuis et al., 2002; Santos-Garcia et al., 2005; Haaland et al., 2009; Trujillo et al., 2011; Mohan and Jagannathan, 2014), and genetic/genomic instability (Ellsworth et al., 2004; Zaky et al., 2008; Giaretti et al., 2012), mitochondrial defects (McDonald et al., 2008; Maggrah et al., 2013; Parr et al., 2013), production of reactive oxygen species (ROS) (Bongers et al., 1995; Jaloszynski et al., 2003; Chan et al., 2017), increased expression of proliferation and apoptosis markers (Birchall et al., 1997; Bascones-Martinez et al., 2013) and epigenetic modifications (Grady, 2005; Lee et al., 2011; Kamiyama et al., 2012; Luo et al., 2014) are also repeatedly found in regions adjacent to malignant tumours from a variety of organs. Whatever the cause of these modifications, from DNA replication errors to mutagenic injuries, the ongoing pre-cancerous field will most likely be composed of a number of genetically different clones, with the fittest one expected to colonise the entire territory over time (Driessens et al., 2012). This process of selection based on fitness comparison is a distinctive trait of cell competition (CC), a phenomenon first observed and characterised in *Drosophila* (Morata and Ripoll, 1975), and then demonstrated to be conserved in mammals (Penzo-Mendez and Stanger, 2014; Di Gregorio et al., 2016).

Competitive interactions are typically triggered when cells with different proliferation rates are found in close proximity: the fittest cells (winners) commit less fit neighbours (losers) to death and overgrow to replace them in the tissue (Levayer and Moreno, 2013, 2016; Tamori and Deng, 2013; Tsuboi et al., 2018). A number of molecules and signalling pathways have to date been found to play a role in CC (Moreno et al., 2002; Tyler et al., 2007; Vincent et al., 2011; Rodrigues et al., 2012; Akai et al., 2018): among these, the MYC protein was shown to be the most powerful inducer of CC (named in this case MYC-Mediated Cell Competition, MMCC) from *Drosophila* to mammals (Johnston, 2014), paving the way to studies that found this process implicated in a number of seemingly distant contexts, from organ development (de la Cova et al., 2004; Moreno and Basler, 2004; Claveria et al., 2013; Sancho et al., 2013; Villa del Campo et al., 2014; Villa Del Campo et al., 2016) to tissue regeneration (Oertel et al., 2006; Gogna et al., 2015; Rosen et al., 2015; Villa Del Campo et al., 2016; Shakiba and Zandstra, 2017), cell stemness (Rhiner et al., 2009; Diaz-Diaz et al., 2017) and cancer (Froldi et al., 2010; Ziosi et al., 2010; Eichenlaub et al., 2016; Suijkerbuijk et al., 2016). Of note, we and others recently demonstrated that MMCC is also active in human cancer cells (Patel et al., 2016; Di Giacomo et al., 2017). MYC upregulation is sufficient as to transform cells into super-competitors (Moreno and Basler, 2004), able to kill and replace suboptimal neighbours, and this capability has opened to speculations about a possible role for MMCC in field cancerisation (Rhiner and Moreno, 2009; Johnston, 2014). MYC family proteins are long investigated for their essential functions in cell physiology and in cancer (Stine et al., 2015); the *Drosophila* genome bears a single *locus* (*diminutive, dm*) encoding the MYC protein, which exerts the same functions as the mammalian orthologues (Gallant, 2013). MYC overexpression in wild-type cells may provoke a series of contradictory responses: on the one hand, it supports cell growth by accelerating biosynthesis, cell metabolism and cell cycle (Evan and Littlewood, 1993; Grewal et al., 2005; Meyer and Penn, 2008); on the other hand, it promotes potentially harmful reactions such as ROS production and genetic instability (Vafa et al., 2002; Greer et al., 2013; Kuzyk and Mai, 2014), and increases propensity to apoptotic cell death (Montero et al., 2008; McMahon, 2014). Cancer cells upregulating MYC are contrariwise protected from untimely death, primarily due to relevant changes in metabolic pathways leading to MYC addiction (Gabay et al., 2014). MYC seems thus to elicit in normal cells a number of biological responses similar to those found in mammalian pre-cancerous fields (Mohan and Jagannathan, 2014). Moreover, MYC upregulation is an early event in human prostate cancer (Gurel et al., 2008), and MYC overexpression is sufficient to transform luminal epithelial cells into pre-malignant derivatives in the mouse prostatic gland (Kim et al., 2009; Iwata et al., 2010). MYC upregulation has also been observed in cytologically normal bronchial epithelial cells of mice with pre-neoplastic lung squamous cell carcinoma lesions (Xiong et al., 2017), and it was reported to initiate gastric tumourigenesis following Hippo pathway deregulation in the pyloric stem cell (Choi et al., 2018). These observations led us to speculate that high MYC levels may be sufficient for an epithelial tissue to become responsive to the effect of second mutations that would otherwise be irrelevant when occurring in a wild-type epithelium.

In *Drosophila*, the tumour suppressor genes (TSGs) are historically subdivided into two classes, called “hyperplastic” and “neoplastic” according to the mutant phenotype (Hariharan and Bilder, 2006), most of which have in time been found to encode different components of the Hippo pathway (Grusche et al., 2010), a highly conserved signalling cascade central in cell growth and organ size modulation (Halder and Johnson, 2011). Broadly speaking, loss-of-function (LOF) mutants of these hyperplastic TSGs (*fat, ft*; *dachsous, ds*; *expanded, ex*; *warts, wts*; and *hippo, hpo*) show a substantial overgrowth of the larval epithelial organs, called imaginal discs (Aldaz and Escudero, 2010), and premature death at the pupal stage (Hariharan and Bilder, 2006), whereas LOF mutants of neoplastic TSGs do not survive beyond embryogenesis (Menut et al., 2007). An exception is made for *scribble* (*scrib*), *discs large* (*dlg*), and *lethal giant larvae* (*lgl*) neoplastic mutants which, given the abundant maternal transcript released into the zygote, survive up to the end of the larval life, showing abnormal growth of the imaginal discs with a complete loss of the epithelial structure (Bilder et al., 2000; Bilder, 2004). In case single mutant cells are created in a wild-type background through clonal analysis techniques (del Valle et al., 2012), those bearing hyperplastic LOF mutations survive and overgrow in the target tissue (Xu et al., 1995; Buratovich and Bryant, 1997; Udan et al., 2003; Maitra et al., 2006), whereas those bearing neoplastic LOF mutations are usually eliminated during development (Agrawal et al., 1995; Enomoto and Igaki, 2011). We and others demonstrated these opposite behaviours are dictated by MMCC: while hyperplastic mutant cells upregulate MYC and behave like winners in the wild-type tissue, killing the surrounding neighbours and growing at their expense (Neto-Silva et al., 2010; Ziosi et al., 2010), neoplastic mutant cells do not upregulate MYC and behave like losers in the context, being themselves out-competed by adjacent wild-type cells (Froldi et al., 2010; Menendez et al., 2010). We previously showed that a MYC-overexpressing background strengthens the super-competitive behaviour of *ft, ds* and *ex* mutant clones, which were found to kill the surrounding cells with increased efficiency and to grow more rapidly, although it did not provide mutant cells with the capability to evolve into a malignant mass (Ziosi et al., 2010).

Here we expanded on previous work by first identifying in *Drosophila* MYC-overexpressing epithelial organs a series of morphological and molecular markers typically found in human pre-cancerous fields. Moreover, we investigated the impact of a MYC-overexpressing background on the cellular phenotypes consequent to mutations in neoplastic TSGs, showing it is in this case sufficient to make mutant cells able to initiate multifocal malignant transformation, a peculiar trait of human pre-neoplastic fields.

## Materials and Methods

### Fly Stocks and Manipulation

The following fly lines were used in the study, built using stocks obtained from the Bloomington Drosophila Stock Center, Indiana: w; UAS-GFP^(Bl-6874)^; hh-Gal4^(Bl-67046)^ – yw, PI3K92E^CAAX(Bl-25908)^ – w; Ubi-GFPnls, FRT40A^(Bl-5629)^/CyO; hh-Gal4^(Bl-67046)^/TM6b – w; l(2)gl^4^ P(neo-FRT)40A^(Bl-36289)^/ In(2-3)Gla,Bc; UAS-HAdm/TM6b – w; Rab5^2^P(neo-FRT) 40A^(Bl-42702)^/In(2-3)Gla,Bc; UAS-HAdm/TM6b. UAS-HAdm on III is a gift of P. Bellosta. Plain genotypes are given for each experiment in the figure legends. For all experiments, flies were kept at 25°C. Larvae were heat-shocked once at 48 ± 4 h AEL in a water bath at 37°C for 10 min and dissected after additional 72 h development.

### Immunofluorescence

Frozen or fresh larvae were prepared for immunofluorescence by standard methods. The following antibodies and dilutions were used: mouse α-MYC (1:5, P. Bellosta); rabbit α-Lgl (1:400, D. Strand); rabbit α-active Caspase 3 (1:100, Cell Signalling Technologies); rabbit α-aPKCζ (1:200, Santa Cruz Biotechnology); rabbit α-pAKT (1:100, Cell Signaling Technologies); rabbit α-PH3 (1:100, Upstate Technology); mouse α-γH2Av (1:30, DSHB); mouse α-dIAP1 (1:100, B. A. Hay); rabbit α-Pc (1:400, Santa Cruz Biotechnology); mouse α-En (1:50, DSHB). Alexa Fluor 555 goat α-mouse and α-rabbit (1:500, Invitrogen) and DyLight 649-conjugated goat α-mouse and α-rabbit (1:750, Jackson ImmunoResearch Laboratories) were used as secondary antibodies. Samples were analysed with a Leica TSC SP2 laser confocal microscope and entire images were processed with Adobe Photoshop software or ImageJ free software from NIH. All the images represent a single confocal stack unless otherwise specified. Image magnification is 400× unless otherwise specified.

### ROS Detection

Larvae were dissected in PBS1X and carcasses were incubated for 30 min at room temperature in PBS1X – DHE (Dihydroethidium, Invitrogen Molecular Probes) at a final concentration of 30 μM in gentle shaking before fixation. Wing discs were immediately imaged under a Nikon 90i wide-field fluorescence microscope.

### Statistical Analysis

**FIGURE 1:**
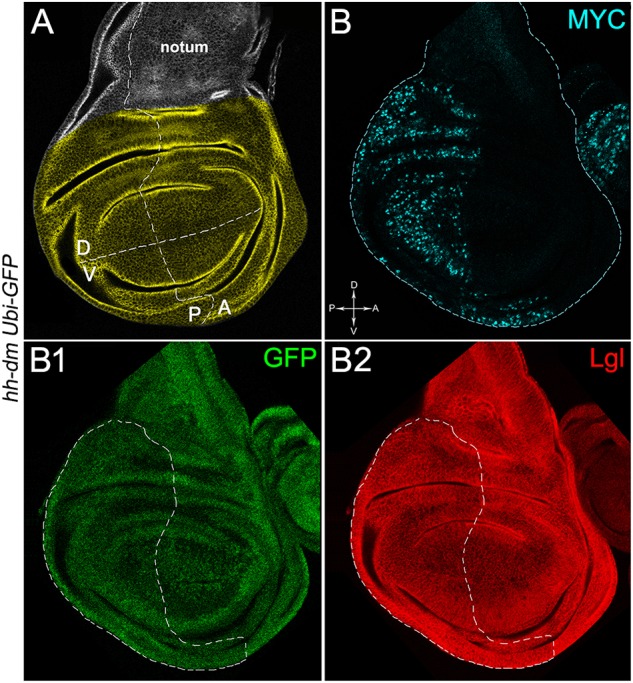
MYC overexpression in the posterior compartment of the wing disc does not cause morphological alterations. **(A)** Representation of an imaginal wing disc from a wild-type late *Drosophila* larva. The Posterior/Anterior (P/A) and the Dorsal/Ventral (D/V) boundaries are indicated by dotted lines. All the measures for this study have been taken in the yellowish area. **(B–B2)** Immunostaining for MYC (**B**, cyan) and Lgl (**B2**, red) on wing discs from late *yw*; *Ubi-GFPnls, FRT40A*/*+*; *hh-Gal4/UAS-dm* larvae. GFP is shown in **B1**. The basic genotype is indicated on the left of the figure panel. P compartments are outlined in **B1,B2**, and disc axes are indicated in **B**. Magnification is 400×.

**FIGURE 2:**
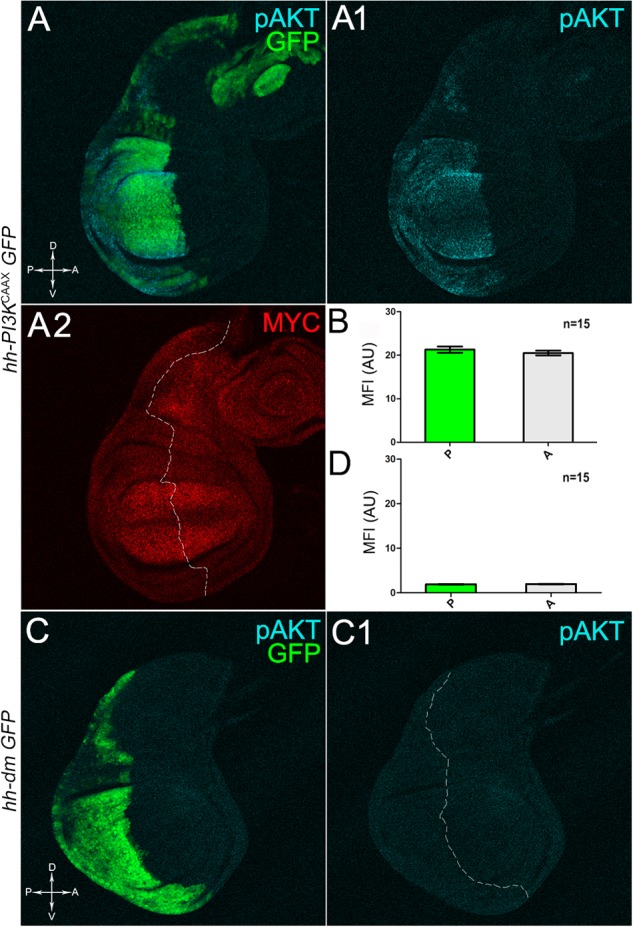
MYC and PI3K^CAAX^ overexpression do not induce reciprocal activation. **(A–A2)** Immunostaining for pAKT (**A,A1**, cyan) and MYC (**A2**, red) on wing discs from late *yw/yw, UAS-PI3K^CAAX^; hh-Gal4, UAS-GFP/+* larvae. **(B)** Graph comparing the Mean Fluorescence Intensity Arbitrary Units (MFI-AU) of MYC staining measured in the P (green bar) and A (grey bar) compartments of 15 wing discs from different larvae. **(C,C1)** Immunostaining for pAKT (cyan) on wing discs from late *yw*; *hh-Gal4, UAS-GFP/UAS-dm* larvae. **(D)** Graph comparing the Mean Fluorescence Intensity Arbitrary Units (MFI-AU) of pAKT staining measured in the P (green bar) and A (grey bar) compartments of 15 wing discs from different larvae. Basic genotypes are indicated on the left of the figure panel. P compartments are outlined in **A2,C1**, and disc axes are indicated in **A,C**. Magnification is 400×.

**FIGURE 3:**
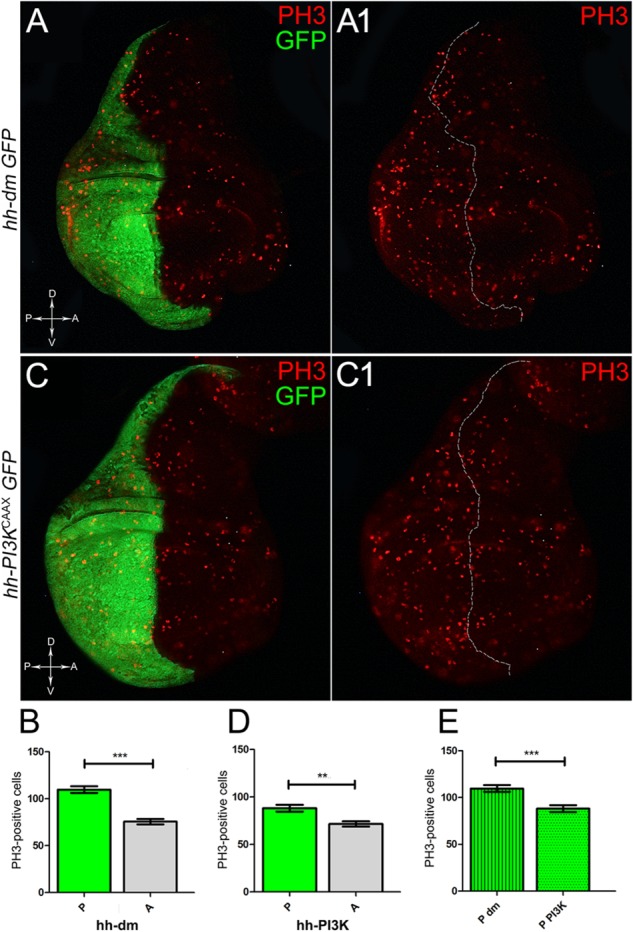
MYC and PI3K^CAAX^ overexpression increases mitotic activity. **(A,A1)** Immunostaining for PH3 (red) on wing discs from late *yw*; *hh-Gal4, UAS-GFP/UAS-dm* larvae. **(B)** Graph comparing the PH3-positive nuclei counted in the P (green bar) and A (grey bar) compartments, ^∗∗∗^*p* ≤ 0.001. **(C,C1)** Immunostaining for PH3 (red) on wing discs from late *yw/yw, UAS-PI3K^CAAX^; hh-Gal4, UAS-GFP/+* larvae. **(D)** Graph comparing the PH3-positive nuclei counted in the P (green bar) and A (grey bar) compartments, ^∗∗^*p* ≤ 0.01. **(E)** Graph comparing the PH3-positive nuclei counted in the P compartments of *yw*; *hh-Gal4, UAS-GFP/UAS-dm* (striped green bar) and *yw/yw, UAS-PI3K^CAAX^; hh-Gal4, UAS-GFP/+* (dotted green bar) larvae, ^∗∗∗^*p* ≤ 0.001. Basic genotypes are indicated on the left of the figure panels and under the graphs. P compartments are outlined in **A1,C1**, and disc axes are indicated in **A,C**. Magnification is 400×.

**FIGURE 4:**
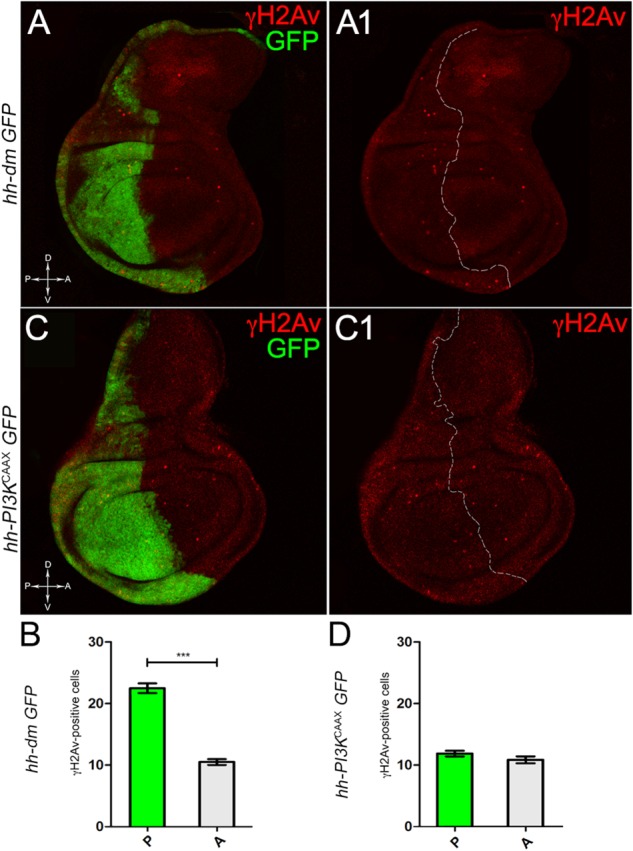
MYC overexpression increases genetic instability. **(A,A1)** Immunostaining for γH2Av (red) on wing discs from late *yw*; *hh-Gal4, UAS-GFP/UAS-dm* larvae. **(B)** Graph comparing the γH2Av-positive foci counted in the P (green bar) and A (grey bar) compartments, ^∗∗∗^*p* ≤ 0.001. **(C,C1)** Immunostaining for γH2Av (red) on wing discs from late *yw/yw, UAS-PI3K^CAAX^; hh-Gal4, UAS-GFP/+* larvae. **(D)** Graph comparing the γH2Av-positive foci counted in the P (green bar) and A (grey bar) compartments. Basic genotypes are indicated on the left of both the figure panels and the graphs. P compartments are outlined in **A1,C1**, and disc axes are indicated in **A,C**. Magnification is 400×.

**FIGURE 5:**
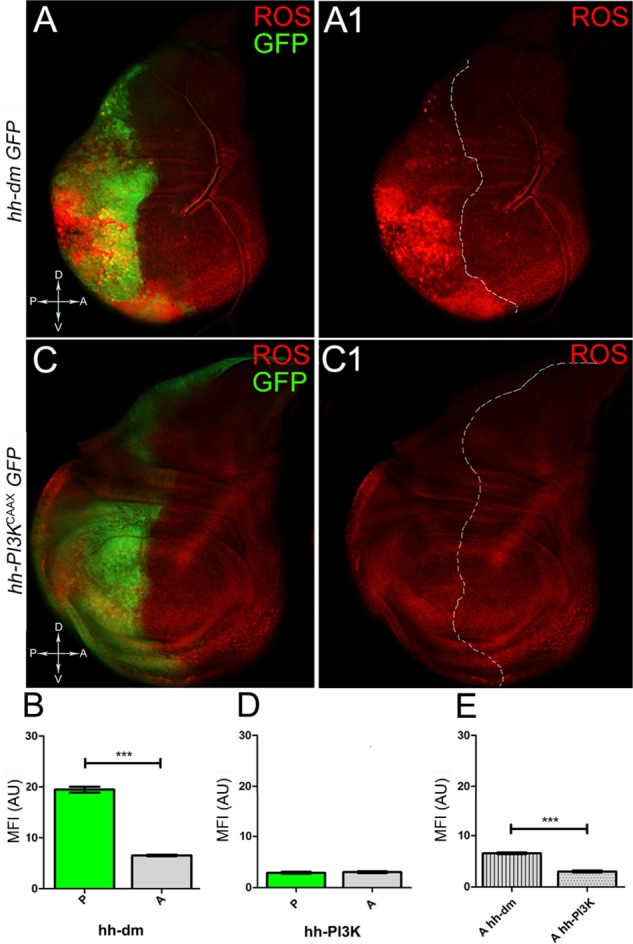
MYC overexpression causes intense ROS production. **(A,A1)** ROS production (red) in wing discs from late *yw*; *hh-Gal4, UAS-GFP/UAS-dm* larvae. **(B)** Graph comparing the Mean Fluorescence Intensity of ROS positivity measured in the P (green bar) and A (grey bar) compartments, ^∗∗∗^*p* ≤ 0.001. **(C,C1)** ROS production (red) in wing discs from late *yw/yw, UAS-PI3K^CAAX^; hh-Gal4, UAS-GFP/+* larvae. **(D)** Graph comparing the Mean Fluorescence Intensity Arbitrary Units (MFI-AU) of ROS positivity measured in the P (green bar) and A (grey bar) compartments. **(E)** Graph comparing the Mean Fluorescence Intensity Arbitrary Units (MFI-AU) of ROS positivity measured in the A compartments of *yw*; *hh-Gal4, UAS-GFP/UAS-dm* (striped grey bar) and *yw/yw, UAS-PI3K^CAAX^; hh-Gal4, UAS-GFP/+* (dotted grey bar) larvae, ^∗∗∗^*p* ≤ 0.001. Basic genotypes are indicated on the left of the figure panels and under the graphs. P compartments are outlined in **A1,C1**, and disc axes are indicated in **A,C**. Magnification is 400×.

**FIGURE 6:**
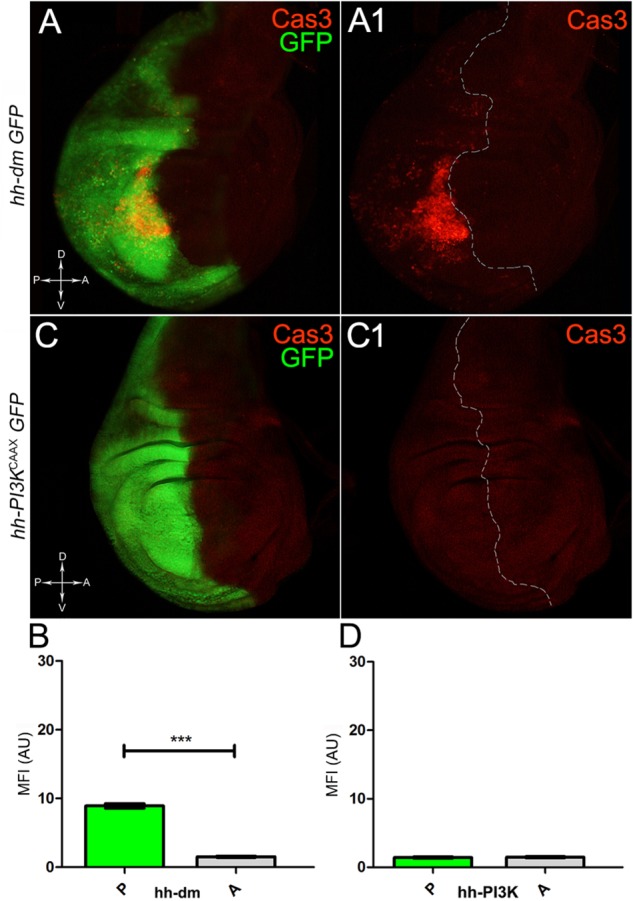
MYC overexpression triggers apoptotic death. **(A,A1)** Immunostaining for Cas3 (red) on wing discs from late *yw*; *hh-Gal4, UAS-GFP/UAS-dm* larvae. **(B)** Graph comparing the Mean Fluorescence Intensity Arbitrary Units (MFI-AU) of Cas3 staining measured in the P (green bar) and A (grey bar) compartments, ^∗∗∗^*p* ≤ 0.001. **(C,C1)** Immunostaining for Cas3 (red) on wing discs from late *yw/yw, UAS-PI3K^CAAX^; hh-Gal4, UAS-GFP/+* larvae. **(D)** Graph comparing the Mean Fluorescence Intensity Arbitrary Units (MFI-AU) of Cas3 staining measured in the P (green bar) and A (grey bar) compartments. Basic genotypes are indicated on the left of the figure panels and under the graphs. P compartments are outlined in **A1,C1**, and disc axes are indicated in **A,C**. Magnification is 400×.

**FIGURE 7:**
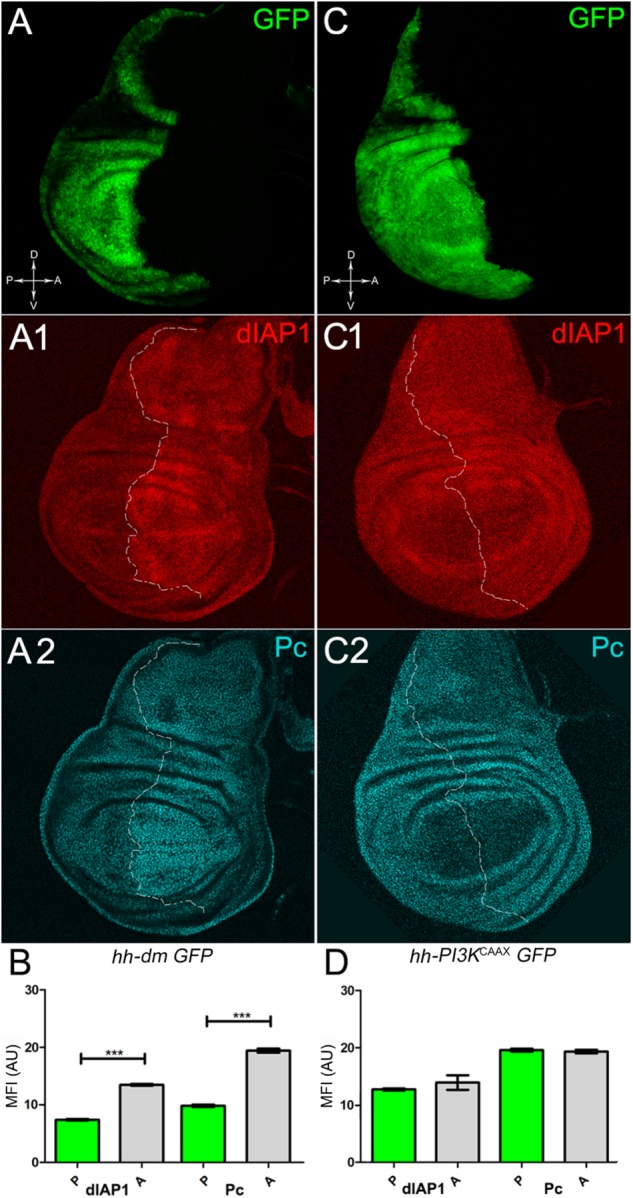
MYC overexpression downregulates survival and epigenetic markers. **(A–A2)** Immunostaining for dIAP1 (**A1**, red) and Pc (**A2**, cyan) on wing discs from late *yw*; *hh-Gal4, UAS-GFP/UAS-dm* larvae. **(B)** Graph comparing the Mean Fluorescence Intensity Arbitrary Units (MFI-AU) of dIAP and Pc staining measured in the P (green bars) and A (grey bars) compartments, ^∗∗∗^*p* ≤ 0.001. **(C–C2)** Immunostaining for dIAP1 (**C1**, red) and Pc (**C2**, cyan) on wing discs from late *yw/yw, UAS-PI3K^CAAX^; hh-Gal4, UAS-GFP/+* larvae. **(D)** Graph comparing the Mean Fluorescence Intensity Arbitrary Units (MFI-AU) of dIAP and Pc staining measured in the P (green bars) and A (grey bars) compartments. Basic genotypes are indicated under the figure panels. P compartments are outlined in **A1,A2,C1,C2**, and disc axes are indicated in **A,C**. Magnification is 400×.

**FIGURE 8:**
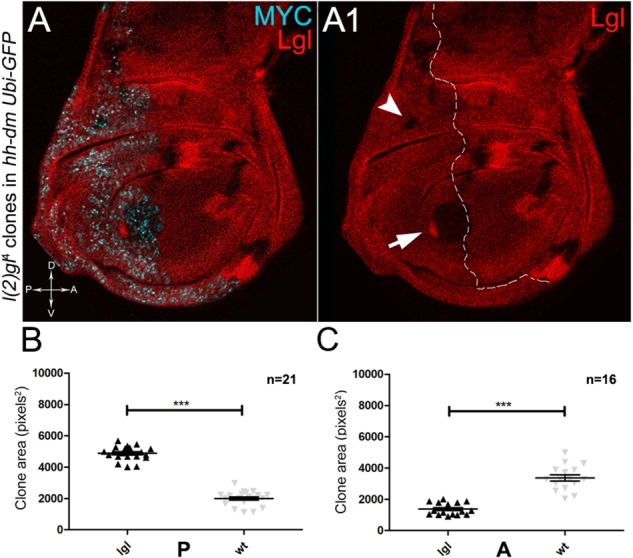
About one/third of *lgl*^-^ clones overgrows at the expense of the surrounding wild-type tissue in a MYC-overexpressing background. **(A,A1)** Immunostaining for Lgl (red) and MYC (cyan) on wing discs from late *w*; *l*(2)*gl*^4^, *FRT40A*/*Ubi-GFPnls, FRT40A*; *hh-Gal4*/*UAS-dm* larvae. The arrow points to a posterior wild-type twin clone and the arrowhead indicates a scattered *lgl*^-/-^ clone with no obvious wild-type twin in the posterior hinge region **(A1)**. **(B,C)** Graphs comparing the average clone area of *lgl*^-/-^ (black triangles, black in the images) and wild-type twins (grey triangles, double red in the images) in the P **(B)** and A **(C)** compartments, ^∗∗∗^*p* ≤ 0.001. The basic genotype is indicated on the left of the figure. The P compartment is outlined in **A1**, and disc axes are indicated in **A**. Magnification is 400×.

**FIGURE 9:**
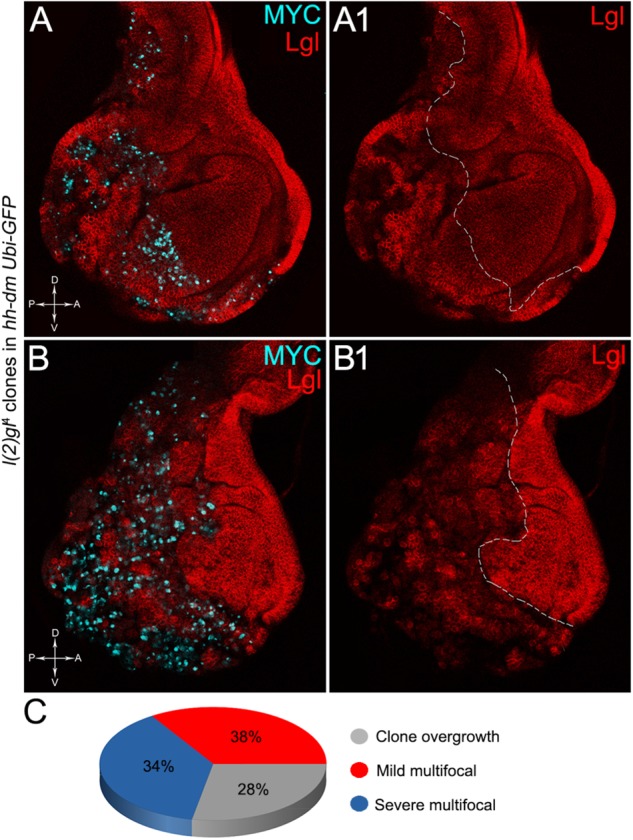
The majority of *lgl*^-/-^ cells forms multifocal nests which colonise a large fraction of the MYC-overexpressing tissue. **(A–B1)** Immunostaining for Lgl (red) and MYC (cyan) on wing discs from late *w*; *l*(2)*gl*^4^, *FRT40A*/*Ubi-GFPnls, FRT40A*; *hh-Gal4*/*UAS-dm* larvae. **(C)** Pie chart illustrating the numerical proportions of overgrown, mild and severe multifocal *lgl*^-/-^ clones found in the P compartment of *w*; *l*(2)*gl*^4^, *FRT40A*/*Ubi-GFPnls, FRT40A*; *hh-Gal4*/*UAS-dm* wing discs. The basic genotype is indicated on the left of the figure panel. P compartments are outlined in **A1,B1**, and disc axes are indicated in **A,B**. Magnification is 400×.

**FIGURE 10:**
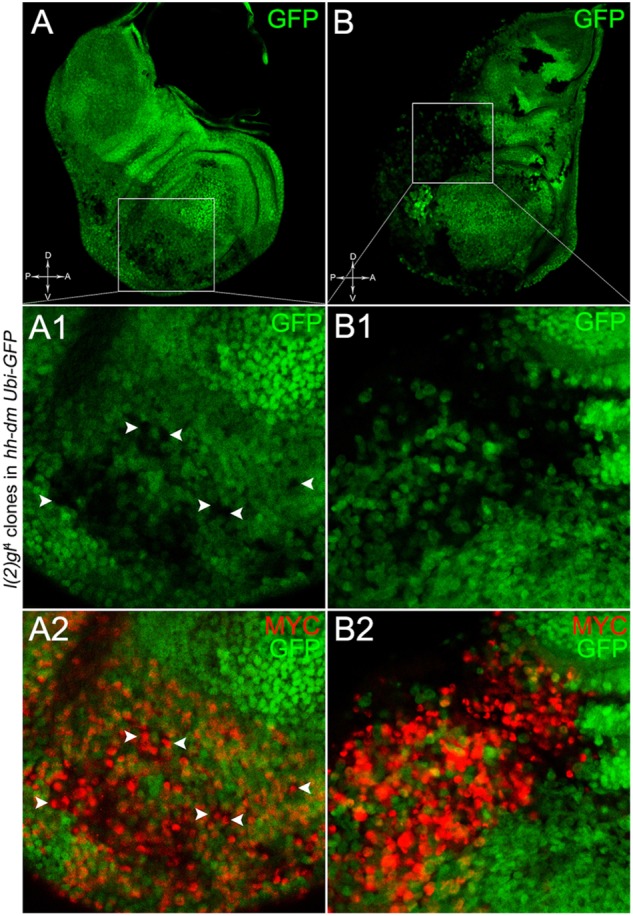
*lgl*^-/-^ cells undergoing severe multifocal growth accumulate MYC protein. Immunostaining for MYC (red) on wing discs from late *w*; *l*(2)*gl*^4^, *FRT40A*/*Ubi-GFPnls, FRT40A*; *hh-Gal4*/*UAS-dm* larvae. In **A1,A2**, arrowheads indicate MYC-accumulating mutant cells. The basic genotype is indicated on the left of the figure panel. Disc axes are indicated in **A,B**. **A1–B2** are magnified views of the squares drawn in **A,B**. Magnification is 400× in **A,B**, 1000× in **A1,A2**, and 1200× in **B1,B2**.

**FIGURE 11:**
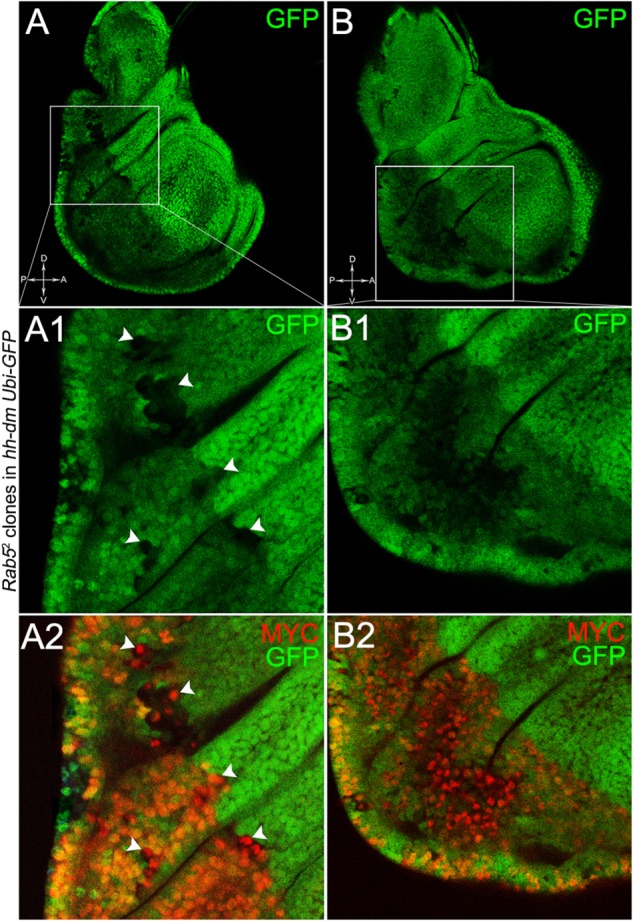
*Rab5*^-/-^ cells show a fully penetrant, multifocal phenotype in a MYC-overexpressing background. Immunostaining for MYC (red) on wing discs from late *w*; *Rab5*^2^, *FRT40A*/*Ubi-GFPnls, FRT40A*; *hh-Gal4*/*UAS-dm* larvae. In **A1,A2**, arrowheads indicate MYC-accumulating mutant cells. The basic genotype is indicated on the left of the figure panel. Disc axes are indicated in **A,B**. **A1–B2** are magnified views of the squares drawn in **A,B**. Magnification is 400× in **A,B**, 1000× in **A1,A2**, and 800× in **B1,B2**.

For the experiments shown in Figures 3–7, the number of wing discs analysed was 15÷25 from different larvae for each sample. For each experiment, the data presented are the average of three biological replicates. Multifocality was assessed on a total of 346 wing discs for *l*(2)*gl*^4^ clones (see Figures 9, 10), and on a total of 146 wing discs for *Rab5*^2^ (see Figure 11). For the experiments shown in Figures 2, 8, 13, the number of discs analysed is indicated. Mean Fluorescence Intensity (MFI) (Figures 2, 5–7), clone area (Figures 8, 13) and positive signals (Figures 3, 4) were calculated by ImageJ free software (NIH) on images captured with a Nikon 90i wide-field fluorescence microscope at a magnification of 200×. All measurements have been taken inside the yellowish area highlighted in Figure 1A. *P*-values were as follows: ^∗∗^*p* ≤ 0.01 and ^∗∗∗^*p* ≤ 0.001. Mean, SEM and the *t*-Student test *p*-value were calculated by using GraphPad Prism software, San Diego, CA, United States.

## Results

### MYC-Overexpressing Tissues Show Several Markers Repeatedly Found in Human Pre-cancerous Fields

Pre-cancerous fields are defined as tissue areas composed of histologically normal but genetically altered cells, shown to be more susceptible than wild-type counterparts to the onset of new mutations, promoting in time the development of multifocal tumours (Slaughter et al., 1953; Dotto, 2014). Since these areas are found to surround primary masses in several epithelial malignancies (Nonn et al., 2009; Zeki et al., 2011; Park et al., 2016), a pre-neoplastic field can be considered, borrowing Paget’s hypothesis, a soil providing “bad seeds” with the capacity to initiate malignant growth, including those that would normally fail. The wide series of aberrations underlying the process of field cancerisation can hardly be attributed to a single cellular event, but deregulation of a gene piloting a number of cell behaviours may greatly favour its formation. MYC represents an excellent candidate, because its misexpression does not account on gene mutation but is rather caused by alterations in many, if not all, signalling pathways (Nussinov et al., 2016). As an example, activated forms of RAS are frequently found in human pre-neoplastic tissues (Braakhuis et al., 2003), and it is known that activated RAS stabilises MYC protein in *Drosophila* (Prober and Edgar, 2002) and mammals (Sears et al., 2000). Stabilised MYC is in turn able to remodulate cell growth and proliferation, metabolism and stress response (Meyer and Penn, 2008). Moreover, a founder cell upregulating MYC could easily expand into a MYC-upregulating field through MMCC (Johnston, 2014). Therefore, MYC could play a causative role both in driving the expansion and in determining the intrinsic characteristics of a pre-cancerous field. To investigate this issue we bypassed field formation, since it is well established that *Drosophila* epithelial cells upregulating MYC eliminate the wild-type neighbours during development and colonise a large fraction of the tissue through MMCC (de la Cova et al., 2004; Moreno and Basler, 2004).

We then took advantage of the UAS-Gal4 binary system (Brand and Perrimon, 1993) to drive MYC overexpression (hereafter referred to as MYC^OV ER^) under the control of the *hedgehog* (*hh*) promoter in the posterior compartment of the wing disc, a *Drosophila* larval epithelial organ (Bryant, 1975). Figure 1A shows the Posterior/Anterior (P/A) and the Dorsal/Ventral (D/V) axes of the larval wing disc, while the yellowish region represents the area subjected to measurements and P vs. A comparisons, being the notum mostly composed of anterior cells (see P/A boundary in the notum region). As can be appreciated in Figure 1B, MYC^OV ER^ is confined to the P compartment (representing the pre-cancerous field), where it does not seem to cause evident alterations in tissue morphology with respect to the A compartment (representing the wild-type field), as noted in Figures 1B1,B2, where a Ubi-GFPnls transgene and the Lgl protein mark cell nuclei and cell membranes, respectively. To demonstrate MYC’s specificity in providing cells with a complex pre-cancerisation signature, we compared the results of each experiment with those obtained following overexpression of a membrane-tethered form of PI3K (PI3K^CAAX^), another potent growth inducer (de la Cova et al., 2004). We first verified if overexpression of the PI3K^CAAX^ transgene (PI3K^CAAX-OV ER^) caused consistent activation of the PI3K/AKT signalling pathway. As noted in Figures 2A,A1, the phosphorylated form of AKT was detected in the P compartment of the wing disc following PI3K^CAAX-OV ER^ (GFP^+^ region in Figure 2A). Moreover, it did not impact MYC endogenous levels (Figure 2A2, the P/A border is outlined), being the MFI of MYC staining statistically comparable in P and A compartments (Figure 2B). Following MYC^OV ER^, the levels of phosphorylated AKT in the P compartment (GFP^+^ region, Figures 2C,C1, the P/A border is outlined) were also comparable to those observed in the A compartment (Figure 2D), confirming that, differently from what has been observed in a previous study (Levayer et al., 2015), in our genetic system and under our working conditions, the two growth inducers do not significantly cross-regulate each other, making it suitable for the successive analyses.

We started by investigating in the MYC^OV ER^ tissue a number of markers characteristic of human pre-neoplastic fields. Since it is known that pre-malignant areas may display a higher proliferative index than normal tissues (Mohan and Jagannathan, 2014), we first checked the mitotic activity of MYC^OV ER^ cells by immunostaining for the phosphorylated histone H3 (PH3), which is known to play a key role during mitosis both in *Drosophila* and mammals (Kamakaka and Biggins, 2005). A mitotic index analysis highlighted a 32% increase of PH3-positive nuclei in MYC^OV ER^ P compartments with respect to their A counterparts (Figures 3A,A1,B), and a 20% increase in the PI3K^CAAX-OV ER^ P vs. A compartments (Figures 3C,C1,D). This result was not unexpected, as PI3K activation plays important roles in cell growth and proliferation (Leevers et al., 1996). The mitotic index of the MYC^OV ER^ tissue was, however, significantly higher than that observed in the PI3K^CAAX-OV ER^ samples, as in the graph reported in Figure 3E. To assess genetic instability, another feature of pre-cancerous fields with obvious mutagenic effects (Bhattacharjee and Nandi, 2016), we used an antibody against the γ variant of the phosphorylated histone H2, which is recognised as the first modification occurring following DNA double strand breaks, resulting in the assembling of multi-protein complexes which attempt to repair DNA damage (Dronamraju and Mason, 2011). As can be seen in Figures 4A,A1, the γH2Av foci (red) in the MYC^OV ER^ P compartment (GFP^+^, outlined in Figure 4A1) were about twice compared to the A compartment (Figure 4B), while they resulted comparable in the P and A compartments of the PI3K^CAAX-OV ER^ samples (Figures 4C,C1,D). Our study continued by evaluating the presence and abundance of ROS in the presumptive pre-cancerous field. As noted in Figures 5A,A1, a strong increase in ROS generation (red) was found in the MYC^OV ER^ P compartment of the wing disc (GFP^+^, outlined in Figure 5A1), quantified as about 20 arbitrary units (AU) MFI vs. the 6.5 AU found in the A compartment (compare green and grey bars in the graph Figure 5B). Contrariwise, no significant differences were found between the P (GFP^+^, outlined in Figure 5C1) and A compartments following PI3K^CAAX-OV ER^, as is appreciable in Figures 5C,C1,D. As MYC^OV ER^ and PI3K^CAAX-OV ER^ samples underwent parallel enzymatic reactions, we could also compare ROS levels in the respective wild-type A compartments, and found that MYC^OV ER^ A compartment showed a twofold ROS increase with respect to the PI3K^CAAX-OV ER^ A compartment (Figure 5E). This was an interesting finding, as ROS are diffusible ions and molecules and they may freely move away from the producing cells, thus expanding MYC’s pre-cancerisation effect to adjacent tissues by a non-autonomous mechanism. In this sense, a recent study demonstrated that, in *Drosophila* epithelial tumours, apoptotic caspases enhance tumour malignancy by generating ROS, which in turn recruit immune cells that signal back to the epithelium to activate cancer pathways (Perez et al., 2017). Although MYC^OV ER^ tissues cannot be compared to overt cancers, similar cell-cell interactions may be at work that cooperate with MMCC to expand the pre-cancerous field. An analysis of apoptotic cell death carried out by immunostaining for the activated form of the effector Caspase 3 (Cas3) revealed that MYC^OV ER^ epithelial cells were highly prone to apoptotic death (see Figures 6A,A1), with about 9 MFI AU in the P compartment vs. 1.5 in the A counterpart, as can be noted in Figure 6B. By contrast, no significant differences were noticed between P and A compartments overexpressing PI3K^CAAX^ (Figures 6C,C1,D). Consistently with Caspase 3 activation, MYC^OV ER^ cells downregulated the anti-apoptotic protein dIAP1 (Wang et al., 1999), as shown in Figure 7A1 (Figure 7A shows the GFP^+^ P compartment), with 7.4 MFI AU in the P vs. 13.5 in the A compartment (Figure 7B); dIAP1 indeed functions by inhibiting the initiator caspase DRONC (Meier et al., 2000) that, in turn, activates the effector caspases. Also in this case, PI3K^CAAX-OV ER^ tissues did not show significant differences in dIAP1 staining between the P and A compartments (Figures 7C,C1,D). Finally, with regard to changes in the epigenetic signature of human pre-neoplastic tissues (Grady, 2005; Lee et al., 2011), we analysed the effect of MYC^OV ER^ on the chromatin modifier Polycomb (Pc), known to shape cellular plasticity through large-scale epigenetic regulation (Klebes et al., 2005). We previously showed that Pc expression is nearly absent in *Drosophila* epithelial cancers (Grifoni et al., 2015). As it is known that Pc and other proteins of the Pc group (PcG) are necessary to MYC auto-repression in *Drosophila* (Goodliffe et al., 2005; Khan et al., 2009), Pc downregulation in overt cancers may help sustain high MYC cellular levels, so allowing it to impact many different phenotypic traits. As can be observed in Figure 7A2, Pc resulted downregulated also in our pre-cancerisation model, with 9.8 vs. 19.4 MFI AU in the P (GFP^+^ in Figure 7A, outlined in 7A2), and A compartments, respectively (Figure 7B). This is consistent with MYC and PcG proteins *trans*-regulation (Benetatos et al., 2014), and since low Pc levels result in a higher chromatin accessibility, this condition would favour additional mutational insults through inappropriate entrance of DNA cleaving enzymes (Zhang et al., 2008). Also in this case, PI3K^CAAX-OV ER^ tissues did not display significant differences compared to the wild-type counterparts (Figures 7C2,D).

Altogether, these results support our hypothesis that high levels of MYC are sufficient as to induce a series of molecular changes, which are likely to turn the affected tissue into a pre-malignant field. Moreover, this ability seems to be specific to MYC, as an active form of the growth inducer PI3K failed to promote significant alterations of the markers analysed.

### Single-Cell Mutations of Neoplastic TSGs Initiate Multifocal Growth in a MYC^OV ER^ Tissue

With the aim to translate the evidence described above into a functional demonstration of MYC^OV ER^’s capacity to establish a pre-malignant condition, we investigated the phenotypic consequences of the induction of second mutations in a MYC^OV ER^ background. We used a genetic model which, through a combination of the UAS-Gal4 (Brand and Perrimon, 1993) and Flp-FRP (Xu and Rubin, 1993) binary systems, allowed us to express MYC in the P compartment and to induce second mutations of interest later in time, so reproducing the temporal sequence that is likely to occur during cancer initiation. The A compartment has been used as a control, to assess the clonal phenotype promoted by the same second mutations in a region carrying endogenous MYC expression.

As described in the Introduction, we previously showed that hyperplastic TSGs (hTSGs) exploit excess MYC to grow more rapidly, but are not able to initiate malignant transformation (Ziosi et al., 2010); we thus aimed at exploring MYC^OV ER^’s effect on the clonal behaviour of neoplastic TSGs (nTSGs). We first analysed the *lethal giant larvae* (*lgl*) mutation. Lgl protein regulates the apical-basal cell polarity in the epithelia (Grifoni et al., 2013); we previously demonstrated its functional conservation from *Drosophila* to humans (Grifoni et al., 2004), and we and others found the human orthologue *HUGL-1* involved in cancers from different organs (Grifoni et al., 2004, 2007; Schimanski et al., 2005; Lu et al., 2009). In the *Drosophila* wing disc, *lgl* mutant cells are unable to grow in a wild-type background, especially in the regions where MYC levels are high, and are eliminated by MMCC (Froldi et al., 2010). In the same wild-type background, MYC^OV ER^ in *lgl* mutant clones rescues them from death and transforms *lgl*^-/-^ cells from losers into super-competitors (Froldi et al., 2010). But what happens to newly formed *lgl*, MYC^OV ER^ cells when they are surrounded by MYC^OV ER^ neighbours? As can be seen in Figure 8, while *lgl*^-/-^ clones were smaller than wild-type twins in the A control compartment of the disc (Figures 8A,A1,C), in the 28% of the wing discs analysed the *lgl*^-/-^ clones growing in the MYC^OV ER^ P compartment appeared significantly larger than the wild-type twins (Figures 8A,A1,B). As an example, the arrow in Figure 8A1 points to a wild-type clone (double red) which appears much smaller than the *lgl* mutant twin (black). In addition, the arrowhead indicates an *lgl* mutant clone in the hinge region of the P compartment with no apparent wild-type twin clone. This suggests that the *lgl* mutant cells have a greater ability to exploit the excess MYC protein than the surrounding neighbours, hence the gain of a competitive advantage over the wild-type tissue. However, the average clone area occupied by the *lgl*^-/-^ cells in this system was about 5000 px^2^, whereas it was found to be around 24000 px^2^ in a previous study where *lgl*^-/-^, MYC^OV ER^ cells were induced in a wild-type background (Froldi et al., 2010), demonstrating the MYC^OV ER^ neighbours exert a competitive pressure against the growth of the *lgl* mutant clones, which translates into a limited capability of *lgl*^-/-^ cells to form large masses in a uniform MYC^OV ER^ field.

The remaining 72% of the wing discs analysed displayed a novel phenotype: the *lgl* mutant tissue grew as a multitude of spots scattered all across the MYC^OV ER^ P compartment. Figure 9 shows two typical samples that we classified as “mild multifocal” (Figure 9A), which represented the 38% of the total samples (Figure 9C), and “severe multifocal” (Figure 9B), which represented the 34% of the total samples (Figure 9C). We classified multifocality as “mild” when the *lgl* mutant clones (black), though colonising a large fraction of the P compartment, did not alter its width (Figure 9A1), and “severe” when the *lgl*^-/-^ cells filled the entire P compartment, which appeared dramatically enlarged (see how the P/A border moved from P to A comparing Figures 9A1,B1). This deep organ alteration suggests a locally invasive, malignant behaviour of these mutant cells that may be favoured by clone confluence during growth, as it is with other tumour models in *Drosophila* (Menendez et al., 2010; Ballesteros-Arias et al., 2014), with MYC protein levels that appeared to increase along with phenotype severity (compare Figures 9A,B). *lgl* mutant cells displayed preferential MYC accumulation, as can be appreciated in Figure 10A2, where arrowheads indicate some of the mutant cells (black, see Figure 10A1) accumulating MYC. Again, the organs displaying larger mutant spots (Figure 10B, squared area) showed an obvious increase in MYC protein levels (Figure 10B2). The most interesting aspect of this model is that it faithfully reproduced a distinctive feature of human pre-cancerous fields, i.e., multifocality (Dotto, 2014). The multifocal phenotype has never been associated with *lgl* mutations in *Drosophila*; therefore, it represents a novel trait acquired by a cell subject to a mutation in the *lgl* nTSG while being part of a MYC^OV ER^ field.

**FIGURE 12:**
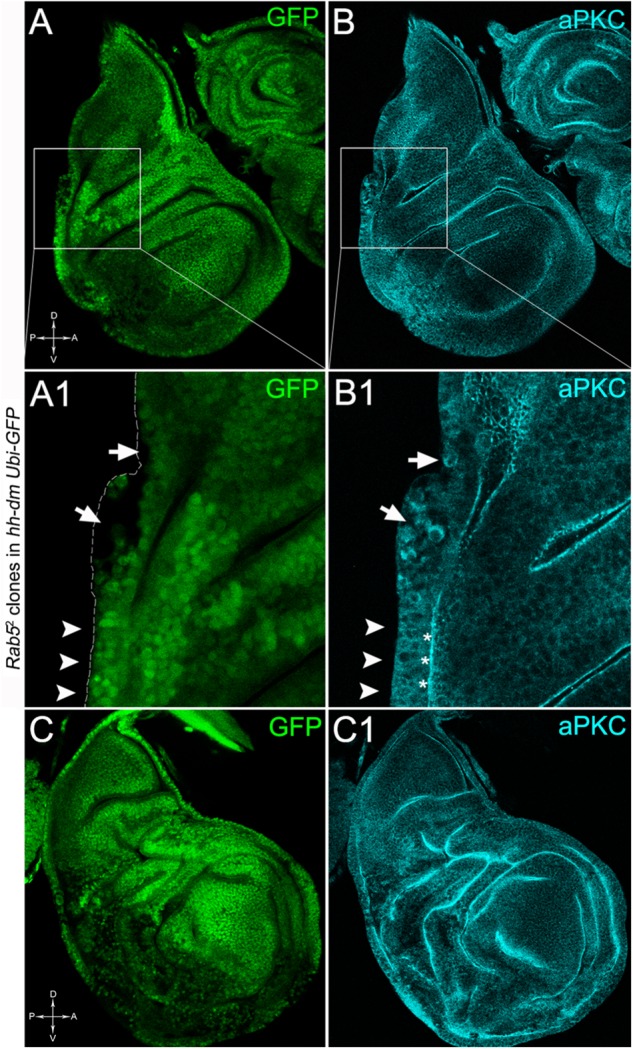
*Rab5*^-/-^ cells show loss of cell polarity in a MYC-overexpressing background. Immunostaining for aPKC (cyan) on wing discs from late *w*; *Rab5*^2^, *FRT40A*/*Ubi-GFPnls, FRT40A*; *hh-Gal4*/*UAS-dm* larvae. In **A1,B1**, arrows indicate mutant cells (black) displaying membrane redistribution of the apical marker aPKC, while the arrowheads point to a normal region of the disc border where the pseudostratified epithelium shows apical aPKC staining (asterisks). The dashed line in **A1** marks the disc outer border. The basic genotype is indicated on the left of the figure panel. Disc axes are indicated in **A,C**. **A1,B1** are magnified views of the squares drawn in **A,B**. Magnification is 400× in **A,B,C,C1** and 1000× in **A1,B1**.

To verify that a MYC^OV ER^ field represented a *bona fide* pre-cancerous area, and that multifocality did not result from a specific interaction between *lgl* and MYC, we induced a LOF mutation of a different nTSG in the MYC^OV ER^ field. Rab5 is an evolutionarily conserved core component of the vesicle trafficking machinery (Lu and Bilder, 2005), implicated in various aspects of human tumourigenesis (Torres and Stupack, 2011; Mendoza et al., 2014). Like *lgl*, entire fly organs mutated for *Rab5* show neoplastic growth (Lu and Bilder, 2005; Vaccari and Bilder, 2009), and *Rab5* mutant cells induced in a wild-type wing disc suffer from cell competition and are eliminated from the organ (Ballesteros-Arias et al., 2014). Using the same clonal system as above, we induced *Rab5* LOF clones in animals whose P compartments overexpressed MYC. As can be seen in Figure 11, the multifocal phenotype was evident also for the *Rab5*^-/-^ cells (Figures 11A,B and respective magnifications A1 and B1). Also in this case, mutant cells showed MYC accumulation (Figures 11A2,B2, arrowheads in A2 indicate some mutant nests accumulating MYC). The 100% of the organs analysed showed a multifocal phenotype, subdivided in 71% mild and 29% severe. Moreover, *Rab5* mutant cells showed loss of apical-basal cell polarity, a central feature of epithelial cancers (Wodarz and Nathke, 2007): in Figure 12, the magnifications in A1 and B1 show a region of the disc outer border where one can appreciate that the normal epithelium (arrowheads in A1 and B1) displays a wild-type localisation of the apical marker atypical PKC (aPKC, cyan, asterisks in B1). On the contrary, the mutant cells in the region indicated by the arrows in A1 and B1 (black in A1) show a redistribution of the polarity marker from the apical side to the entire cell cortex, together with aberrant, three-dimensional growth. In Figures 12C,C1, the impairment of aPKC expression (cyan) is evident across the entire MYC^OV ER^ P compartment. This characteristic is consistent with Rab5 function: the endocytic trafficking is indeed essential in the maintenance of cell polarity, and mutations in genes involved in endocytosis provoke the expansion of cell’s apical domain (Shivas et al., 2010).

Altogether, this evidence indicated that MYC overexpression in an epithelial tissue is sufficient to promote multifocal malignant lesions following single-cell mutations of different nTSGs.

**FIGURE 13:**
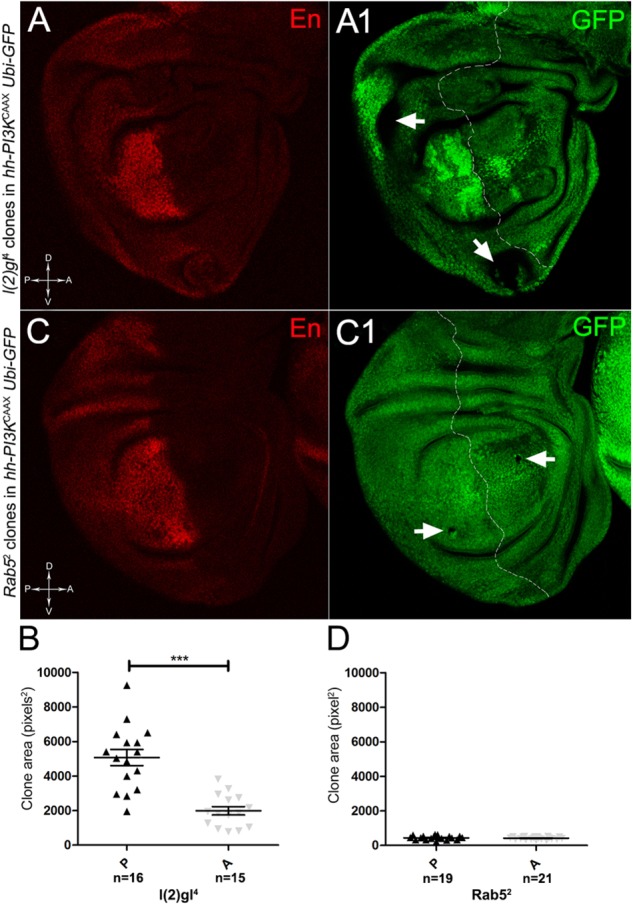
*lgl*^-/-^ and *Rab5*^-/-^ cells do not display multifocal growth in a PI3K^CAAX^-overexpressing background. **(A,A1)** Immunostaining for En (red) on wing discs from late *w/yw, UAS-PI3K^CAAX^; l*(2)*gl*^4^, *FRT40A*/*Ubi-GFPnls; hh-Gal4/+* larvae. The arrows in **A1** indicate the mutant clones (black). **(B)** Graph comparing the average clone area of *lgl*^-/-^ clones in the P (black triangles) and A (grey triangles) compartments, ^∗∗∗^*p* ≤ 0.001. **(C,C1)** Immunostaining for En (red) on wing discs from late *w/yw, UAS-PI3K^CAAX^; Rab5*^2^, *FRT40A*/*Ubi-GFPnls; hh-Gal4/+* larvae. The arrows in **C1** indicate the mutant clones (black). **(D)** Graph comparing the average clone area of *Rab5*^-/-^ clones in the P (black triangles) and A (grey triangles) compartments. Basic genotypes are indicated on the left of the figure panel. The P compartment is outlined in **A1,C1**, and disc axes are indicated in **A,C**. Magnification is 400×.

To assess if multifocality may be considered a trait arising from specific properties conferred by the MYC field to the mutant cells, we repeated the same experiments as above in a PI3K^CAAX-OV ER^ territory. Using the same system as above, we first analysed *lgl* mutant behaviour. In Figures 13A,A1, we can observe *lgl* mutant clones (GFP^-^, indicated by the arrows) in the PI3K^CAAX^ P compartment (marked in red by En staining in A). They are located outside the central region of the disc where, instead, we observed the presence of wild-type clones (GFP^2+^), indicating that mutant twins were eliminated by MMCC. Therefore, despite the over-expression of PI3K^CAAX^, *lgl*^-/-^ clones continue to die in this area of the wing discs where MYC is normally highly expressed (see Figure 2A2). A statistical analysis of the clone area in the P and A compartments showed that *lgl*^-^*^/^*^-^ mutant clones were significantly larger in P, with an average size of about 5000 px^2^, compared to A, where they displayed an average size of about 2000 px^2^ (Figure 13B). The most important observation was, however, the total absence of multifocal growth. We then analysed the behaviour of the *Rab5*^2^ mutation in a PI3K^CAAX^ background. In Figures 13C,C1, a wing disc is shown where small mutant clones of comparable size are present in both compartments (black, arrows in Figure 13C1). In Figure 13D, the graph indicates that the mutant clones do not show significant differences in size between the P and A compartments. Finally, as it was for *lgl*, no multifocal growth was observed in all the *Rab5* samples analysed.

These latter findings indicate that MYC confers on cells mutant for different nTSGs the ability to grow in multiple foci dispersed all across the modified territory. This seems to be a specific characteristic of MYC, as the growth inducer PI3K did not promote this peculiar phenotype. MYC upregulation emerges from our study as an excellent candidate to foster field cancerisation, by inducing a complex pre-cancerisation molecular signature able to provide cells hit by non-competitive mutations with the ability to initiate carcinogenesis.

## Discussion

Field cancerisation is studied in the effort to understand if essential events recur in tumour initiation that may help develop early therapeutic interventions. It is now recognised that many types of cancers start from cells owing some, but not all, phenotypic traits necessary for malignancy, and those traits may result from various mutagenic insults, on the basis of which the most performant cells are selected for clonal expansion (Curtius et al., 2018). This process may be driven by cell competition, which is intensively studied both in *Drosophila* (Merino et al., 2016) and mammals (Di Gregorio et al., 2016). In this context, we focused our attention on MMCC, a process based on steep differences in MYC levels in confronting cells, which ultimately favour the expansion of high MYC-expressing cells at the expense of the less fit neighbours (Grifoni and Bellosta, 2015). Given the broad implication of MYC protein in human cancers (Gabay et al., 2014), its myriad functions inside the cell (Dang et al., 2006) and its regulation at both the transcriptional and post-transcriptional levels by a number of signalling pathways (Nussinov et al., 2016), it seems an excellent candidate to pioneer field cancerisation (Moreno, 2008).

To address this question, we first investigated the cellular responses to MYC overexpression (MYC^OV ER^) in the imaginal wing disc, a *Drosophila* epithelial tissue widely used to model development, cell competition and cancer (Herranz et al., 2016). We found that MYC^OV ER^ was *per se* sufficient to activate a series of cellular behaviours consistent with the formation of a pre-neoplastic field, such as ROS production, genetic instability, changes in apoptotic and proliferation activity and alteration of epigenetic markers. Moreover, we showed that these cellular responses were not elicited by a MYC’s generic pro-growth function, as an active form of the powerful growth inducer PI3K was not able to induce similar phenotypes, except a mild pro-proliferative effect. High MYC levels seem rather to prime field cancerisation by triggering a cascade of molecular changes that cooperate in taking cells a step closer to malignancy.

This *bona fide* pre-cancerous tissue was then tested for the ability to initiate tumourigenesis following mutations in neoplastic TSGs (nTSGs). We previously studied the effects of MYC^OV ER^ on three hyperplastic TSGs (hTSGs) owing to the Hippo signalling pathway: *ds, ft* and *ex*, and found that mutant clones grew more rapidly while killing the MYC^OV ER^ wild-type neighbours with higher efficiency, but they did not show any signs of malignancy (Ziosi et al., 2010). We and others demonstrated that most hTSGs upregulate MYC (Neto-Silva et al., 2010; Ziosi et al., 2010), hence their competitive capability, while some nTSGs downregulate MYC, hence their elimination from the tissue (Froldi et al., 2010). It is also recognised that the behaviour of both hTSGs and nTSGs depends on tissue’s MYC levels (Froldi et al., 2010; Neto-Silva et al., 2010; Ziosi et al., 2010): in a uniform background, as with our model, mutant behaviour should rather be dictated by the intrinsic features of the given mutation.

In the *Drosophila* wing disc, wild-type cells hit by nTSGs mutations are usually irrelevant: they are indeed eliminated rapidly or contribute to the tissue without overgrowing (Froldi et al., 2010; Ballesteros-Arias et al., 2014). The same mutations induced in a MYC^OV ER^ field were rather capable to initiate multifocal, three-dimensional growth accompanied by loss of apical-basal cell polarity and aberrant tissue architecture. This was convincing evidence that MYC upregulation was sufficient as to establish a specific, complex pre-cancerisation signature, which predisposes the tissue to undergo malignant multifocal growth following certain second mutations.

Our findings lay the basis for future studies focused on early tumourigenesis. These studies are as essential as difficult: while understanding the very first phases of cancer is mandatory to conceive novel preventive and therapeutic interventions, investigations carried out in complex systems may lead to discouraging results. In this sense, the use of a genetically amenable animal model may greatly help dissect and dismantle the intricate networks implicated in cancer initiation.

## Author Contributions

MS and DG conceived and designed the study. MS, CG, and SP performed the experiments. DG and DdB analysed the experimental data. SDG performed the statistical analysis. AP and DG wrote the manuscript. All authors contributed to manuscript revision and read and approved the submitted version.

## Conflict of Interest Statement

The authors declare that the research was conducted in the absence of any commercial or financial relationships that could be construed as a potential conflict of interest.
